# Supplementary material for: Mechanisms governing the pioneering and redistribution capabilities of the non-classical pioneer PU.1
Source: Nat Commun. 2020 Jan 21;11:402. doi: 10.1038/s41467-019-13960-2 (PMC6972792; doi:10.1038/s41467-019-13960-2)
Supplement: Supplementary file 7 — Source data [file 41467_2019_13960_MOESM7_ESM.zip › Source_Data/FigureS2/FigureS2B_MotifScanOutput/KmeansCluster10/homerResults/motif11.similar.html]

motif11

## Information for motif11

A
T
G
C
G
C
T
A
A
T
G
C
A
C
G
T
C
A
G
T
A
G
T
C
G
A
T
C
A
G
C
T
G
A
T
C
C
G
T
A
T
C
G
A
C
G
T
A
  
Reverse Opposite:  

A
C
G
T
A
G
C
T
A
C
G
T
C
T
A
G
T
C
G
A
C
T
A
G
A
C
T
G
G
T
C
A
C
G
T
A
T
A
C
G
C
G
A
T
A
T
C
G
  

|  |  |
| --- | --- |
| p-value: | 1e-66 |
| log p-value: | -1.539e+02 |
| Information Content per bp: | 1.712 |
| Number of Target Sequences with motif | 212.0 |
| Percentage of Target Sequences with motif | 5.71% |
| Number of Background Sequences with motif | 507.0 |
| Percentage of Background Sequences with motif | 1.15% |
| Average Position of motif in Targets | 97.8 +/- 41.4bp |
| Average Position of motif in Background | 103.9 +/- 63.3bp |
| Strand Bias (log2 ratio + to - strand density) | 0.1 |
| Multiplicity (# of sites on avg that occur together) | 1.01 |
| Motif File: | file (matrix) reverse opposite |

### Similar de novo motifs found

|  |  |  |  |  |  |  |  |
| --- | --- | --- | --- | --- | --- | --- | --- |
| Rank | Match Score | Redundant Motif | P-value | log P-value | % of Targets | % of Background | Motif file |
| 1 | 0.914 | G C T A A T G C G C A T A C G T A G T C G T A C A T C G A G T C G T C A T G C A C G T A | 1e-59 | -136.122727 | 10.24% | 3.75% | motif file (matrix) |
| 2 | 0.833 | A C G T A C G T T A G C C G T A A T C G A G C T A C G T A G T C A G T C A G C T A G T C G C T A C T G A G T C A | 1e-35 | -81.712983 | 2.24% | 0.31% | motif file (matrix) |
| 3 | 0.869 | C A T G T A G C C G T A A C T G A G C T C A G T A G T C A G T C A G T C G A C T G T C A C T G A G T C A | 1e-26 | -61.757814 | 1.51% | 0.18% | motif file (matrix) |
